# Function-preserving plasma kinetic enucleation of the prostate with bladder neck and 12 o'clock urethral mucosal preservation for large-volume benign prostatic hyperplasia: a single-center retrospective case series

**DOI:** 10.3389/fsurg.2026.1959580

**Published:** 2026-10-14

**Authors:** Wenshuai Yan, Jie Jia, Yanming Huo, Jixue Gao, Feng Wang, Hongxiong Song

**Affiliations:** 1 Department of Urology, Affiliated Hospital of Yan'an University, Yan'an, Shaanxi, China; 2 Department of Gynecology, Affiliated Hospital of Yan'an University, Yan'an, Shaanxi, China

**Keywords:** benign prostatic hyperplasia, bladder neck, ejaculation, transurethral resection of the prostate, urethral mucosa, urinary incontinence

## Abstract

**Background:**

Large-volume benign prostatic hyperplasia (BPH) poses a significant surgical challenge, as conventional transurethral resection yields suboptimal tissue removal efficiency and high rates of ejaculatory dysfunction. While endoscopic enucleation techniques (e.g., HoLEP and PKEP) offer improved efficacy, the systematic preservation of functional anatomical structures — particularly the bladder neck and anterior urethral mucosa — remains inadequately addressed in current practice.

**Objective:**

To evaluate the safety, efficacy, and functional outcomes of function-preserving plasma kinetic enucleation of the prostate (FP-PKEP) — a modified technique incorporating systematic preservation of the bladder neck circular fibers and the 12 o'clock urethral mucosal strip — in patients with large-volume BPH.

**Methods:**

This single-center, retrospective case series included 52 consecutive patients with large-volume BPH (prostate volume >80 mL, International Prostate Symptom Score [IPSS] > 20, maximum urinary flow rate [Qmax] < 10 mL/s) who underwent FP-PKEP between January 2025 and December 2025. The primary outcome was the change in IPSS at 3 months postoperatively; secondary outcomes included Qmax, post-void residual urine volume (PVR), quality of life (QoL) score, perioperative safety parameters, and functional preservation outcomes (urinary continence and ejaculatory function at 6 months).

**Results:**

All 52 patients (mean age 67.8 ± 7.2 years; mean prostate volume 93.7 ± 15.2 mL) successfully underwent FP-PKEP without conversion. Mean operative time was 72.5 ± 15.8 min; postoperative hemoglobin decrease was 9.8 ± 4.2 g/L; catheterization duration was 2.8 ± 0.9 days; and hospital stay was 4.2 ± 1.3 days. At 3 months, IPSS decreased from 26.1 ± 3.0 to 6.8 ± 2.6 (*P* < 0.001), and Qmax increased from 7.2 ± 1.6 to 21.2 ± 4.5 mL/s (*P* < 0.001). Transient stress urinary incontinence occurred in 4 patients (7.7%), all requiring no more than one pad per day and resolving within 1 month without surgical intervention. Among the 16 patients who were sexually active before surgery, antegrade ejaculation was preserved in 14 (87.5%) and suspected retrograde ejaculation was recorded in 2 (12.5%). No clinically apparent cases of permanent urinary incontinence, bladder neck contracture, or urethral stricture were observed during the 6-month follow-up.

**Conclusions:**

In this uncontrolled case series, FP-PKEP showed a favorable safety profile and achieved significant relief of bladder outlet obstruction, with promising early urinary continence and ejaculatory outcomes. The systematic preservation of the bladder neck and 12 o'clock urethral mucosa warrants further evaluation in prospective randomized controlled trials, as these preliminary findings are exploratory and hypothesis-generating.

## 1 Introduction

Benign prostatic hyperplasia (BPH) is one of the most common urological disorders among middle-aged and elderly men; its histological prevalence is approximately 50% in men over the age of 60 and reaches 90% by age 80 (1). Bladder outlet obstruction (BOO) resulting from BPH can lead to progressively worsening lower urinary tract symptoms (LUTS), significantly impairing patients’ quality of life and closely correlating with serious complications such as recurrent urinary tract infections, acute urinary retention, and upper urinary tract dysfunction (2). With the accelerating global aging of the population, the proportion of patients with large-volume BPH (typically defined as a prostate volume >80 mL) continues to rise, creating an urgent need for surgical interventions that offer long-term efficacy and manageable complication profiles (3).

Transurethral resection of the prostate (TURP) has long been considered the gold standard for the surgical treatment of BPH (4). However, TURP has inherent limitations when managing large-volume prostates: tissue resection efficiency decreases as the gland volume increases, and prolonged operative times raise the risks of hemorrhage and transurethral resection syndrome (TURS) (5). Furthermore, during the resection of hyperplastic tissue, it is difficult to precisely preserve the circular smooth muscle of the bladder neck and the urethral mucosa at the prostatic apex, resulting in postoperative retrograde ejaculation rates as high as 60%–75% and short-term urinary incontinence rates of 10%–20% (6). These functional complications have become key factors limiting patient satisfaction following surgery.

In recent years, endoscopic enucleation of the prostate (EEP)—a technique based on the concept of anatomical enucleation and encompassing procedures such as holmium laser enucleation of the prostate (HoLEP) and plasmakinetic enucleation of the prostate (PKEP)—has achieved tissue removal efficiency comparable to that of open surgery, thanks to its technical advantage of completely stripping the gland along the surgical capsule (7). Meta-analyses have confirmed that EEP is comparable or superior to TURP in improving IPSS and Qmax, while also resulting in less intraoperative blood loss and shorter hospital stays (8, 9). Due to lower equipment costs and reliable hemostasis, PKEP holds unique value for implementation in resource-limited clinical settings (10).

However, as the philosophy of prostate surgery shifts from merely pursuing “maximal resection” toward “precise functional preservation,” the research focus for EEP has moved from surgical efficiency to postoperative functional recovery (11). Anatomical studies have elucidated the importance of preserving two key functional structures (1): the circular smooth muscle fibers of the bladder neck, which form the “proximal gate” that closes the bladder outlet during ejaculation—their integrity directly determines the ability for antegrade semen expulsion (12); and (2) the smooth muscle fibers deep to the urethral mucosa at the 12 o'clock position of the prostatic apex, which are continuous with the anterior wall of the external urethral sphincter and form a suspension-support structure for the anterior urethral wall—preserving this structure is considered a key anatomical basis for promoting early recovery of urinary continence (13, 14). A HoLEP technique involving the preservation of anterior urethral mucosa, as described by Fujisaki et al. (15), reduced the postoperative urinary incontinence rate to 4.1%; similarly, Qiu et al. (16) achieved comparable urinary continence outcomes using a strategy that preserved the urethral mucosa from the bladder neck to the prostatic apex during HoLEP. However, clinical studies simultaneously and systematically implementing both bladder neck preservation and 12 o'clock urethral mucosa preservation within the framework of PKEP remain very limited.

Based on the aforementioned anatomical evidence and insights from previous studies, this study proposes a “function-preserving plasmakinetic enucleation of the prostate” (FP-PKEP) technique. This approach involves the systematic preservation of the circular fibers of the bladder neck and the strip of urethral mucosa at the 12 o'clock position, while adhering to the principles of anatomical enucleation to completely remove the hyperplastic glandular tissue. We hypothesize that FP-PKEP can reduce the incidence of early postoperative urinary incontinence and retrograde ejaculation while maintaining equivalent improvements in voiding function. A retrospective analysis was conducted on the perioperative and short-term follow-up data of 52 patients with large-volume BPH, aiming to provide preliminary evidence regarding the clinical value of this modified surgical technique.

## 2 Materials and methods

### 2.1 Study design

This study was a single-center retrospective case series, designed and reported in accordance with the Strengthening the Reporting of Observational Studies in Epidemiology (STROBE) guidelines (17).

### 2.2 Patients

All consecutive patients with large-volume BPH who underwent FP-PKEP at the Department of Urology, Affiliated Hospital of Yan'an University, between January 2025 and December 2025 were screened for eligibility. During the study period, 187 patients were diagnosed with large-volume BPH (prostate volume >80 mL); 78 of them were treated with FP-PKEP by the senior surgical team, whereas the remaining patients underwent TURP, conventional PKEP, or HoLEP, or received conservative treatment. After initial screening and eligibility assessment of the 78 patients treated with FP-PKEP, 26 patients were excluded and 52 patients formed the final study cohort (Figure 1). As this was a retrospective case series of consecutive patients, no *a priori* sample size calculation was performed; the sample size reflects the number of eligible patients who were treated with FP-PKEP during the study period. Details of patient selection and follow-up are presented in Figure 1.

**Figure 1:**
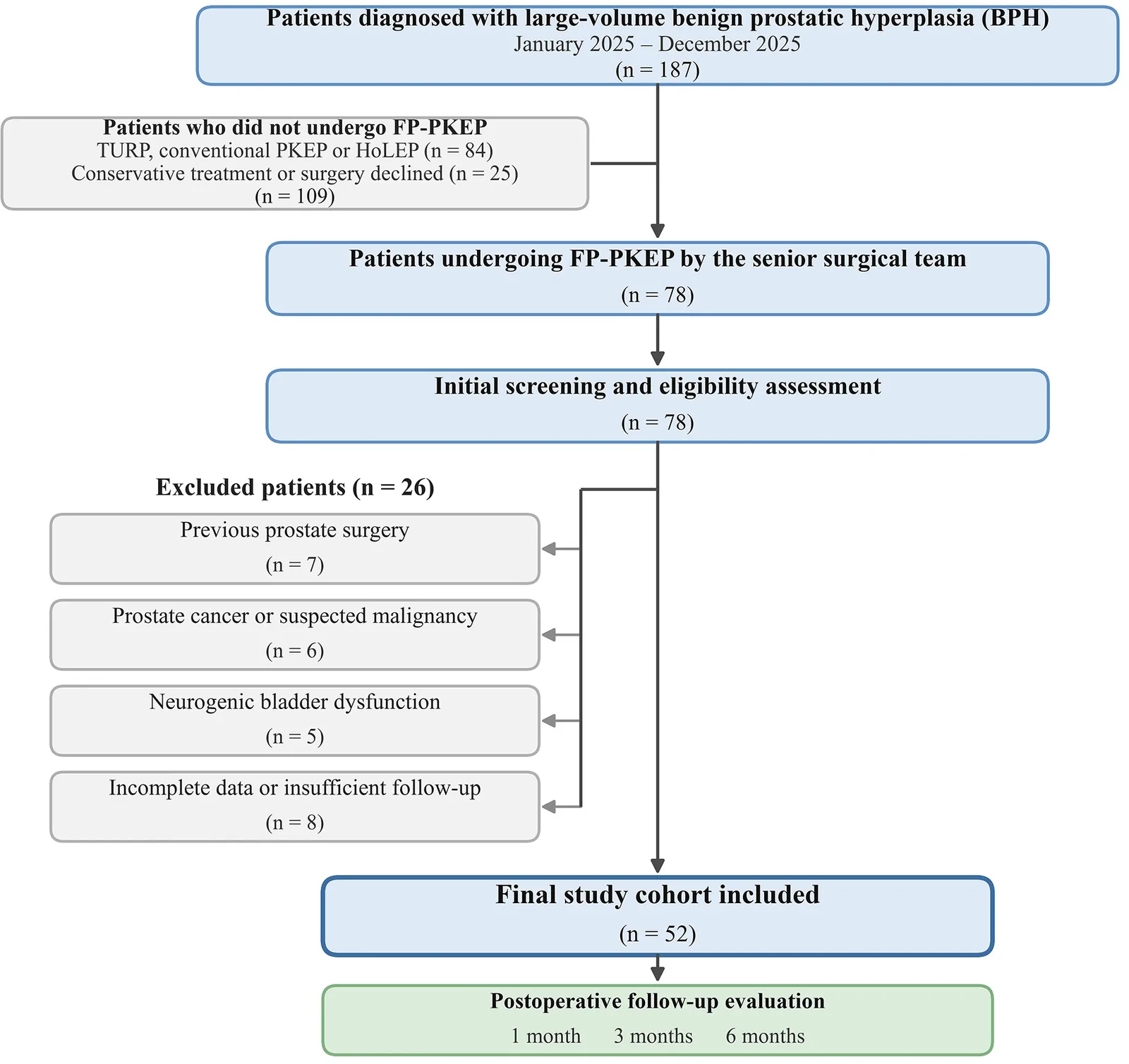
Flow diagram of patient selection and follow-up. BPH, benign prostatic hyperplasia; FP-PKEP, function-preserving plasma kinetic enucleation of the prostate; HoLEP, holmium laser enucleation of the prostate; PKEP, plasma kinetic enucleation of the prostate; TURP, transurethral resection of the prostate.

Inclusion criteria: (1) age ≥50 years; (2) met the clinical diagnostic criteria for BPH, including a clinical diagnosis of benign prostatic obstruction based on medical history, digital rectal examination, and transrectal ultrasonography, as outlined in the “Guidelines for the Diagnosis and Treatment of Urological Diseases in China” (18); (3) prostate volume >80 mL as measured by transrectal ultrasound; (4) IPSS >20 and Qmax <10 mL/s; (5) unsatisfactory response to ≥3 months of pharmacological therapy or patient refusal of long-term medication; (6) undergoing prostate surgery for the first time.

Exclusion criteria: (1) histologically confirmed prostate cancer on transrectal prostate needle biopsy, or high clinical suspicion of malignancy based on digital rectal examination, serum prostate-specific antigen (PSA) level, or imaging findings; (2) a history of open prostatectomy, TURP, or other transurethral prostate surgery; (3) concurrent neurogenic bladder, severe urethral stricture, or bladder neck contracture; (4) coagulation dysfunction (International Normalized Ratio [INR] > 1.5 or platelet count <50 × 10⁹/L); (5) incomplete clinical data or insufficient follow-up (less than 6 months).

Preoperative assessment of prostate cancer risk followed the institutional diagnostic pathway. Serum total prostate-specific antigen (PSA) was measured in all patients. In patients with a total PSA between 4 and 10 ng/mL, the free-to-total PSA (f/t PSA) ratio was determined, and multiparametric magnetic resonance imaging (MRI) of the prostate with transrectal ultrasound-guided prostate biopsy was performed when the f/t PSA ratio was below 0.16; patients with a total PSA above 10 ng/mL underwent multiparametric MRI and prostate biopsy directly. Patients with histologically confirmed prostate cancer were excluded.

### 2.3 Surgical technique (FP-PKEP)

All procedures were performed by the same chief urologist who had completed at least 100 PKEP cases. Continuous epidural anesthesia or general anesthesia was administered, and patients were placed in the lithotomy position. A bipolar plasmakinetic resection system was used (27F continuous-flow sheath; cutting power 160 W, coagulation power 80 W) with isotonic saline as the irrigation fluid. Following enucleation, the detached adenoma was morcellated and removed using a tissue morcellator. The core procedural steps of FP-PKEP are shown in Figure 2.

**Figure 2:**
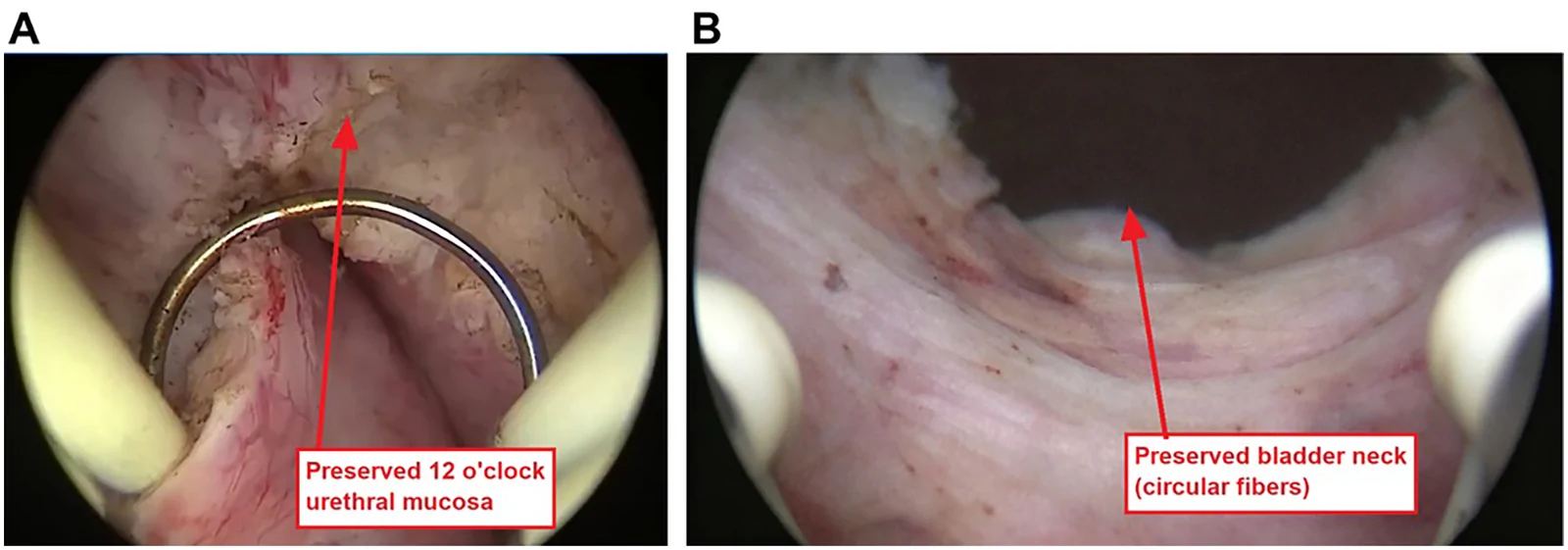
Key functional-preserving steps of FP-PKEP. **(A)** The 12 o'clock urethral mucosal strip is preserved *in situ* as a vault-shaped suspensory structure after bilateral lobe enucleation, providing anterior urethral support that may contribute to early recovery of urinary continence. **(B)** The bladder neck circular fibers are preserved upon completion of median lobe enucleation, which is proposed to contribute to the passive bladder outlet closure mechanism relevant to ejaculatory function.

#### 2.3.1 Identification of anatomical landmarks and establishment of the enucleation plane

After inserting the resectoscope, the morphology of the bladder neck, the location of the ureteral orifices, and the degree of hyperplasia in the various prostatic lobes are first observed to assess the severity of bladder outlet obstruction (BOO). The surgical capsule plane of the prostate is identified proximal to the verumontanum; the urethral mucosa is then incised down to the capsule plane at the 5 o'clock and 7 o'clock positions using a plasma resection loop, creating the entry point for subsequent retrograde enucleation.

#### 2.3.2 Strategy for bladder neck preservation

Retrograde enucleation of the median lobe is performed along the established capsule plane. Upon reaching the level of the bladder neck, the characteristic circular, palisade-like smooth muscle fiber bundles serve as the boundary for preservation; at this point, advancement of the resection loop toward the bladder is halted. Instead, the connection between the median lobe and the bladder neck is freed via blunt dissection using the resectoscope sheath, ensuring the integrity of the circular muscle bundles and the overlying transitional epithelium against thermal injury from the resection loop. This maneuver aims to preserve the passive closure mechanism of the bladder outlet, providing an anatomical basis for the retention of postoperative ejaculatory function.

#### 2.3.3 Strategy for preserving urethral mucosa at the 12 o'clock position

Retrograde enucleation of the left and right lobes begins proximal to the verumontanum and proceeds gradually along the surgical capsule plane toward the bladder neck, adhering to the aforementioned preservation principles upon arrival at the bladder neck. When addressing the anterior commissure (i.e., the 12 o'clock position extending from the prostatic apex to the bladder neck), the hyperplastic glandular tissue is pushed toward the bladder cavity using the resectoscope sheath, clearly exposing the arch-like strip of urethral mucosa that runs along the anterior (12 o'clock) wall of the prostatic urethra from the bladder neck orifice to the level of the verumontanum. Subsequently, the plasma resection loop is used to sharply divide the connecting fibers between the adenoma and the mucosa while keeping close to the adenoma capsule, allowing the mucosal strip to remain suspended *in situ*. This step represents a key technical distinction between this procedure and the traditional three-lobe PKEP method (in which the anterior commissure mucosa is typically resected along with the glandular tissue); it aims to maintain the suspensory support of the anterior wall of the external sphincter and the mechanical continuity of the anterior urethral wall.

#### 2.3.4 Glandular processing, hemostasis, and postoperative management

After the three glandular lobes were fully mobilized and pushed into the bladder cavity, the tissue morcellator was exchanged to morcellate and extract the tissue. Following a careful inspection of the surgical bed, precise point-like hemostasis was achieved using bipolar electrocoagulation; circumferential coagulation or the formation of extensive eschar was avoided in the area of the preserved bladder neck and the region near the external sphincter. At the conclusion of the procedure, an F20 three-way silicone Foley catheter was placed transurethrally, with the balloon inflated with 30 mL of fluid for intravesical fixation, and connected to a continuous isotonic saline bladder irrigation system. The irrigation rate was adjusted based on the color of the drainage fluid, and irrigation was discontinued once the drainage fluid became clear.

### 2.4 Outcomes

Primary outcome measure: change in IPSS from baseline at 3 months postoperatively. Change in IPSS was chosen as the primary outcome measure of this analysis because adequate relief of bladder outlet obstruction is the fundamental objective of any enucleation procedure and must be demonstrated before functional preservation can be considered meaningful. The functional preservation outcomes (urinary continence and ejaculatory function) were treated as secondary, exploratory endpoints, as the study was not powered to evaluate them. Because this was a retrospective analysis, the outcome measures were defined at the analysis stage rather than prospectively registered; this is acknowledged in Section 4.6.

Secondary outcome measures: (1) changes in Qmax, PVR, and QoL scores at 1, 3, and 6 months postoperatively; (2) perioperative safety parameters: operative time, decrease in hemoglobin level 24 h postoperatively, duration of continuous bladder irrigation, duration of catheterization, and length of postoperative hospital stay; (3) functional preservation outcomes. Urinary continence was assessed by structured questioning about leakage and by the number of pads used per day. Transient stress urinary incontinence (TSUI) was defined as any reported leakage of urine on coughing, sneezing, or physical exertion requiring no more than one pad per day, and was distinguished from urgency and mixed urinary incontinence. Patients were classified as sexually active when, on the basis of the preoperative clinical interview, they reported regular or recent sexual activity and were able to provide information on ejaculation before and after surgery; only these patients were eligible for the exploratory assessment of ejaculatory function. Ejaculatory function was assessed by patient-reported clinical interview at each follow-up visit, in which patients were asked about sexual activity, orgasmic sensation, and whether semen was emitted from the urethral meatus during orgasm. No validated instrument (for example, the Male Sexual Health Questionnaire-Ejaculatory Dysfunction Short Form [MSHQ-EjD-SF]) and no post-ejaculatory urinalysis were used; retrograde ejaculation was therefore recorded as clinically suspected rather than objectively confirmed (4). Adverse events occurring during the perioperative period and within 6 months postoperatively, recorded according to the Clavien-Dindo classification system.

### 2.5 Follow-up

Baseline data were extracted from the electronic medical record system, including demographic characteristics, comorbidities, prostate volume (measured via transrectal ultrasound), serum PSA, IPSS, QoL scores, and urodynamic parameters (Qmax, PVR). Perioperative data were prospectively recorded from surgical and nursing records. Postoperative follow-up was conducted in the outpatient clinic at 1, 3, and 6 months post-surgery, comprising assessments of IPSS, QoL, Qmax, PVR, urinary continence status, and ejaculatory function. Follow-up consisted of clinical assessment and symptom scoring and did not include protocolized cystoscopy; therefore, the absence of bladder neck contracture or urethral stricture was determined by clinical and functional assessment rather than by direct endoscopic visualization. Continence status was recorded at each scheduled follow-up visit; recovery within the first postoperative month was therefore documented at the 1-month visit, at which patients reported when they had regained full control. Patients with incomplete data or insufficient follow-up were excluded from the analysis (Figure 1); consequently, complete 1-, 3-, and 6-month data were available for all 52 patients in the final cohort.

### 2.6 Statistical analysis

Statistical analysis was performed using SPSS 26.0 (IBM Corp., Armonk, NY, USA). Continuous variables were first assessed for normality using the Shapiro–Wilk test; data following a normal distribution were expressed as mean ± standard deviation (x¯ ± s) and compared between preoperative and postoperative time points using the paired t-test, while data not following a normal distribution were expressed as median (interquartile range) [M (Q1, Q3)] and compared using the Wilcoxon signed-rank test. Categorical variables were expressed as frequencies (percentages). Comparisons of repeated measurements at multiple postoperative time points (1, 3, and 6 months) vs. baseline were conducted using repeated measures ANOVA or the Friedman test, depending on the data distribution. All statistical tests were two-sided, with a significance level of *α* = 0.05.

## 3 Results

### 3.1 Baseline characteristics

The 52 patients ranged in age from 51 to 84 years, with a mean age of (67.8 ± 7.2) years; prostate volume ranged from 80 to 138 mL, with a mean of (93.7 ± 15.2) mL; and the median serum PSA level was 4.1 (2.6, 6.5) ng/mL. Comorbidities included hypertension in 31 cases (59.6%), type 2 diabetes in 15 cases (28.8%), coronary heart disease in 9 cases (17.3%), and chronic obstructive pulmonary disease in 16 cases (30.8%). Preoperative IPSS was (26.1 ± 3.0) points and Qmax was (7.2 ± 1.6) mL/s, both indicating severe bladder outlet obstruction. Baseline patient characteristics are detailed in Table 1.

**Table 1:** Baseline characteristics of the study cohort (*n* = 52).

Variable	Value
Age (years), mean ± SD (range)	67.8 ± 7.2 (51–84)
Prostate volume (mL), mean ± SD	93.7 ± 15.2
PSA [ng/mL, median (Q1, Q3)]	4.1 (2.6, 6.5)
IPSS, mean ± SD	26.1 ± 3.0
QoL score, median (Q1, Q3)	5 (4, 5)
Qmax (mL/s), mean ± SD	7.2 ± 1.6
PVR [mL, median (Q1, Q3)]	115 (70, 175)
Hypertension, *n* (%)	31 (59.6)
Type 2 diabetes, *n* (%)	15 (28.8)
Coronary artery disease, *n* (%)	9 (17.3)
COPD, *n* (%)	16 (30.8)

IPSS, International Prostate Symptom Score; QoL, quality of life; Qmax, maximum urinary flow rate; PVR, post-void residual urine volume; PSA, prostate-specific antigen; COPD, chronic obstructive pulmonary disease. Continuous variables are presented as mean ± SD or median (Q1, Q3); categorical variables as *n* (%).

### 3.2 Perioperative outcomes

All 52 patients successfully underwent FP-PKEP, achieving a surgical success rate of 100% (52/52); there were no cases requiring conversion to open surgery or a change in the surgical procedure. Perioperative data are summarized in Table 2. The operative time was (72.5 ± 15.8) min, and the decrease in hemoglobin level 24 h postoperatively was (9.8 ± 4.2) g/L; no patients received perioperative red blood cell transfusions. The duration of continuous bladder irrigation was (18.5 ± 5.2) h, the duration of urethral catheterization was (2.8 ± 0.9) days, and the postoperative hospital stay was (4.2 ± 1.3) days.

**Table 2:** Perioperative parameters (*n* = 52).

Parameter	Mean ± SD (range)
Operative time (min)	72.5 ± 15.8 (48–105)
Hemoglobin decrease (g/L)ᵃ	9.8 ± 4.2 (4–18)
Bladder irrigation time (h)	18.5 ± 5.2 (10–28)
Catheterization duration (days)	2.8 ± 0.9 (2–5)
Postoperative hospital stay (days)	4.2 ± 1.3 (3–7)

ᵃAbsolute difference between preoperative and 24-h postoperative hemoglobin. No patient received perioperative blood transfusion.

### 3.3 Improvement in urinary function (primary and secondary efficacy outcomes)

All patients completed follow-up assessments at 1, 3, and 6 months postoperatively; changes in functional parameters are shown in Table 3. At 3 months postoperatively, the primary outcome measure—the IPSS score—decreased from a baseline of 26.1 ± 3.0 to 6.8 ± 2.6 (paired t-test: t = 36.42, *P* < 0.001), representing a reduction of 19.3 points (74.0%). Qmax increased from a baseline of 7.2 mL/s to 21.2 mL/s (t = 21.93, *P* < 0.001), an increase of 14.0 mL/s (194.4%). PVR decreased from a baseline of 115 (70, 175) mL to 15 (8, 25) mL (Z = −6.33, *P* < 0.001). Continuous improvement was observed across all parameters between 1 and 3 months postoperatively, and further improvement was recorded at 6 months (IPSS 5.4 ± 2.2; Qmax 22.4 ± 4.3 mL/s; PVR 12 6, 20 mL; QoL 1 0, 2).

**Table 3:** Comparison of functional outcomes before and after FP-PKEP (*n* = 52).

Variable	Preoperative	1 month	3 months	6 months	Statisticᵃ	*P* value
IPSS, mean ± SD	26.1 ± 3.0	10.2 ± 3.5	6.8 ± 2.6	5.4 ± 2.2	t = 36.42	<0.001
QoL, median (Q1, Q3)	5 (4, 5)	2 (1, 3)	1 (1, 2)	1 (0, 2)	Z = −6.42	<0.001
Qmax (mL/s), mean ± SD	7.2 ± 1.6	16.5 ± 3.8	21.2 ± 4.5	22.4 ± 4.3	t = 21.93	<0.001
PVR [mL, median (Q1, Q3)]	115 (70, 175)	28 (15, 45)	15 (8, 25)	12 (6, 20)	Z = −6.33	<0.001

IPSS, International Prostate Symptom Score; QoL, quality of life; Qmax, maximum urinary flow rate; PVR, post-void residual urine volume. ᵃPaired t-test (t) for normally distributed variables; Wilcoxon signed-rank test (Z) for non-normally distributed variables. *P* values represent comparisons between preoperative and 6-month values.

### 3.4 Functional preservation outcomes

#### 3.4.1 Recovery of urinary continence

Transient stress urinary incontinence occurred in 4 cases (7.7%) postoperatively; all presented as minor leakage during coughing or sneezing requiring no more than one pad per day, and none reported urgency or mixed urinary incontinence. Following pelvic floor muscle training (Kegel exercises), all four patients regained full voluntary urinary control within one month post-surgery, without the need for pharmacological or surgical intervention; recovery was documented at the 1-month visit (Section 2.5). At the 6-month follow-up, all patients (100%) reported satisfactory urinary continence, and no clinically apparent case of persistent urinary incontinence was observed. Routine cystoscopy was not performed during follow-up; therefore, the absence of bladder neck contracture and urethral stricture was based on clinical and functional assessment rather than on direct endoscopic visualization, and subclinical or early lesions cannot be excluded.

#### 3.4.2 Ejaculatory function

Among the 52 patients, 16 (30.8%) were classified as sexually active before surgery on the basis of the preoperative clinical interview (Section 2.4); the remaining 36 patients were not sexually active, were unable to provide information on ejaculation, or were not assessed, and were therefore not eligible for the exploratory ejaculatory-function analysis. Eligibility required the patient to report antegrade ejaculation before surgery; accordingly, all 16 evaluable patients had antegrade ejaculation preoperatively. Follow-up assessment at 6 months post-surgery showed that 14 patients (87.5%) maintained antegrade ejaculation, while 2 patients (12.5%) were recorded as having clinically suspected retrograde ejaculation on the basis of patient report (absence of semen emission from the urethral meatus during orgasm, or a marked reduction in semen volume); post-ejaculatory urinalysis was not performed, so retrograde ejaculation was not objectively confirmed. Given the small number of evaluable patients, this finding should be regarded as exploratory.

### 3.5 Adverse events

Localized perforation of the prostatic surgical capsule occurred intraoperatively in 2 cases (3.8%); the perforations were limited in extent (<1 cm) and did not involve the rectal or bladder walls, thus not hindering the completion of the surgery, and all patients followed a standard postoperative recovery course. Early postoperative adverse events included mild gross hematuria in 3 cases (5.8%), which resolved after extending bladder irrigation for 12–24 h, and transient difficulty voiding in 1 case (1.9%), which resolved after re-insertion of the urinary catheter for 3 days followed by successful removal. According to the Clavien-Dindo classification (19), all the aforementioned postoperative adverse events were Grade I. Because the Clavien-Dindo system is primarily intended to classify postoperative complications, the two intraoperative capsular perforations are reported separately above and were not assigned a Clavien-Dindo grade. During the 6-month follow-up period, no clinically apparent cases of bladder neck contracture, urethral stricture, delayed hemorrhage, or need for re-intervention were observed; however, as cystoscopy was not routinely performed, subclinical or early strictures cannot be fully excluded. A summary of complications is presented in Table 4.

**Table 4:** Adverse events and functional preservation outcomes (*n* = 52).

Event	*n* (%)
Intraoperative complications	
Capsular perforation	2 (3.8)
Bladder injury	0
Blood transfusion	0
Early postoperative complications (Clavien-Dindo grade I)	
Transient urinary incontinence	4 (7.7)
Gross hematuria	3 (5.8)
Dysuria	1 (1.9)
Late complications (at 6 months)	
Permanent urinary incontinence	0
Bladder neck contracture	0
Urethral stricture	0
Delayed hemorrhage	0
Ejaculatory function (*n* = 16)^a^	
Antegrade ejaculation preserved	14 (87.5)
Retrograde ejaculation (clinically suspected)	2 (12.5)

ᵃEjaculatory function assessed only in patients who were sexually active preoperatively and completed 6-month follow-up. Clavien-Dindo classification per Dindo et al., Ann Surg (2004) (19).

## 4 Discussion

### 4.1 Key findings

This study evaluated the preliminary outcomes of a modified PKEP technique (FP-PKEP)—centered on the concept of functional preservation—in 52 patients with large-volume BPH. The key findings are summarized as follows: (1) FP-PKEP achieved improvements in voiding function comparable to those reported in the literature for HoLEP/PKEP; at 3 months postoperatively, the IPSS decreased by 19.3 points (74.0%) and Qmax increased by 14.0 mL/s (194.4%), indicating that the preservation of the bladder neck and the mucosa at the 12 o'clock position did not compromise the adequacy of obstruction relief; (2) the incidence of transient postoperative urinary incontinence was 7.7%, with all cases resolving completely within one month, which is below the rates generally reported in the literature, although this comparison is indirect and the present study had no control group; (3) among the 16 patients who were sexually active preoperatively, 87.5% retained antegrade ejaculation, and the rate of clinically suspected retrograde ejaculation (12.5%) was lower than that commonly reported after traditional TURP and after EEP techniques that do not employ selective functional preservation; however, this sub-analysis included only 16 patients, and cross-study comparisons cannot substitute for a direct controlled comparison. Taken together, these preliminary results are consistent with the concept of “precise functional preservation based on anatomical enucleation,” but they remain exploratory and hypothesis-generating given the uncontrolled design of the study.

### 4.2 Mechanisms of voiding function improvement and comparison with literature

Adequate removal of hyperplastic glandular tissue is the anatomical prerequisite for relieving bladder outlet obstruction (BOO) and achieving sustained improvement in voiding function. A 7-year follow-up of a randomized controlled trial (RCT) by Gilling et al. (20) demonstrated that improvements in IPSS and Qmax following HoLEP were sustained for up to 7 years, with a long-term reoperation rate of less than 5%. The efficacy data for FP-PKEP at 3 months postoperatively in this study (IPSS 6.8 points, Qmax 21.2 mL/s) are consistent with that RCT and other reports on PKEP series (8–10), confirming that adequate relief of mechanical obstruction can be achieved even when incorporating functional preservation maneuvers. It is worth emphasizing that the core of FP-PKEP is not to reduce the extent of glandular resection—the preservation of anterior lobe mucosa merely involves optimizing the enucleation pathway—but rather to avoid unnecessary structural damage while ensuring complete enucleation. This concept aligns closely with the idea of “selective tissue-sparing enucleation” summarized in the systematic review by Pang et al. (11).

### 4.3 Preservation of urinary continence: the anatomical significance of the urethral mucosa at the 12 o'clock position

The mechanisms underlying post-prostatectomy urinary incontinence involve multiple factors, including direct injury to the external urethral sphincter, thermal or traction injury to the innervating nerves, and the loss of urethral supporting structures (13). In this study, the rate of transient urinary incontinence (7.7%) was lower than the rates reported by Coman et al. (21) for HoLEP (16.0%) and by Tricard et al. (22) for HoLEP in cases of very large-volume BPH (>150 mL). However, because these data derive from different study populations and because the present study had no control group, this difference should be interpreted as an indirect observation rather than as evidence of superiority; in our cohort, continence was restored in all cases within one month.

From a procedural perspective, we hypothesize that the preservation of an arch-like strip of urethral mucosa at the 12 o'clock position may be a key technical factor facilitating the early recovery of urinary continence. The smooth muscle fibers deep to this mucosal strip not only form an anatomical continuum with the anterior wall of the external sphincter but also achieve a state of “self-suspension” once the dissection of the left and right lateral lobes is complete—meaning that, following the loss of lateral support, this arched strip becomes the sole suspension pivot for the anterior wall. We propose that this mechanism may be analogous to the biomechanical principles of mid-urethral slings used in surgery for stress urinary incontinence, although the mechanical environments differ significantly. Studies by Fujisaki et al. (15) on anterior urethral mucosa-sparing HoLEP and by Lin et al. (14) on the preservation of the anterior fibromuscular stroma of the prostate support, from different perspectives, the hypothesis that “structural integrity of the anterior wall may contribute to faster recovery of urinary continence.”

### 4.4 Preservation of ejaculatory function: evidence and limitations regarding bladder neck preservation

Retrograde ejaculation is one of the most common long-term complications following TURP; the rate of retrograde ejaculation associated with traditional TURP can reach 60%–80% (6). A systematic review by Marra et al. (6) indicated that the disruption of bladder neck closure function is the primary mechanism underlying retrograde ejaculation; thus, any technical modification that minimizes injury to the circular smooth muscle of the bladder neck may reduce the incidence of this complication. The rate of clinically suspected retrograde ejaculation in our cohort was 12.5% (2/16), which was lower than that associated with traditional TURP, as well as the rates reported by Habib et al. (23) for bipolar plasmakinetic resection of the prostate (BPRP) and by Bhandarkar et al. (24) for PKEP.

However, such cross-study comparisons should be interpreted with caution. Significant heterogeneity exists across studies regarding the definition of “history of sexual activity,” the methods used to assess ejaculatory function (e.g., validated questionnaires such as the MSHQ-EjD versus simple questioning), and the timing of follow-up. In our cohort, patients with a history of sexual activity accounted for only 30.8% (16/52) of the total; the small sample size may have amplified random fluctuations. Furthermore, the assessment of ejaculatory function relied on subjective reporting, introducing a risk of information bias. Consequently, conclusions drawn from these comparisons should be viewed as exploratory findings rather than definitive results.

### 4.5 Safety evaluation

All postoperative adverse events in our cohort were classified as Clavien-Dindo grade I, and no severe complications of grade III or higher occurred; the two intraoperative capsular perforations were not graded according to Clavien-Dindo, as this system applies primarily to postoperative complications. Two cases of capsular perforation (3.8%) were detected intraoperatively, were localized, and did not affect the surgical procedure; this rate is lower than the 5%–10% range reported in PKEP series (10, 21). We hypothesize that this is attributable to the extensive use of the blunt pushing technique with the sheath in FP-PKEP, which reduces the likelihood of accidental capsular injury caused by the resection loop. The postoperative decrease in hemoglobin (9.8 g/L) was minimal, and no blood transfusions were required, consistent with the inherent hemostatic advantages of plasma-based technology. Notably, no clinically apparent cases of bladder neck contracture were observed in this cohort; this is likely attributable to the intraoperative adherence to the principle of “avoiding circumferential electrocoagulation in the functional preservation zone,” as circumferential thermal injury is considered a primary trigger for postoperative fibrotic scarring of the bladder neck (23).

### 4.6 Study limitations

This study has the following limitations, which should be fully considered when interpreting the results.

First (study design): This was a single-center retrospective case series with a low level of evidence (Oxford Centre for Evidence-Based Medicine [CEBM] Level 4) and lacked a concurrent control group undergoing traditional PKEP for direct comparison. Consequently, the inference that FP-PKEP is superior to the traditional procedure regarding urinary continence and ejaculatory function is hypothesis-generating rather than confirmatory. In addition, because of the retrospective design, the outcome measures were defined at the analysis stage rather than prospectively registered.

Second (sample size): The sample size was only 52 cases, with a subgroup of just 16 patients having a history of sexual activity and completed follow-up for ejaculatory function. For low-incidence events (such as bladder neck contracture and urethral stricture, reported in the literature at 1%–5%), the study lacked sufficient statistical power to reliably estimate their incidence rates. The same limitation applies to the assessment of urinary continence: with only 52 patients, the study was similarly underpowered to estimate reliably the true incidence of persistent urinary incontinence or of other rare complications.

Third (follow-up duration): The median follow-up period was only 6 months, which is insufficient to assess the long-term stability of voiding function, long-term reoperation rates, or trends in ejaculatory function over time (as some patients may experience delayed functional changes). In addition, cystoscopy was not performed as part of routine follow-up, so subclinical or early urethral strictures and bladder neck contractures may have gone undetected.

Fourth (assessment of ejaculatory function): The assessment of ejaculatory function did not utilize validated standardized tools (such as the Male Sexual Health Questionnaire-Ejaculatory Dysfunction Short Form [MSHQ-EjD-SF]) nor objective parameter measurements (e.g., ejaculate volume, sperm count, or post-ejaculatory urine analysis for sperm); thus, the true incidence of retrograde ejaculation may have been underestimated or overestimated. Similarly, urinary continence was assessed by pad use and clinical questioning rather than with a validated instrument such as the International Consultation on Incontinence Questionnaire-Urinary Incontinence Short Form (ICIQ-UI SF), so the impact of incontinence on quality of life was not quantified.

Fifth (confounding bias): As an observational study, the influence of patient selection bias and follow-up information bias on the results cannot be ruled out. All procedures were performed by a single senior surgeon who had already completed more than 100 conventional PKEP procedures before this series, and the preservation manoeuvres are performed along the same anatomical planes as standard PKEP; the potential influence of a learning curve specific to the preservation technique is therefore likely to be limited, although it cannot be formally quantified in a single-operator series.

Sixth (surgical parameters and histopathology): enucleated tissue weight, enucleation and morcellation efficiency, and postoperative residual prostate volume were not systematically recorded, and the histopathological findings of the morcellated specimens, including the incidence of incidental prostate cancer, were not collected for this analysis. Because tissue-preserving modifications could in theory affect the completeness or efficiency of adenoma removal, and because the rate of incidental prostate cancer in this cohort was not determined, these remain important reporting gaps that should be addressed in future studies.

Seventh (preoperative assessment of prostate cancer risk): preoperative assessment followed the institutional pathway described in Section 2.2. The detailed results of multiparametric MRI and of prostate biopsy were not systematically tabulated for this analysis, and individual deviations from the pathway cannot be entirely excluded.

### 4.7 Clinical significance and future directions

The core technical concept of FP-PKEP—establishing a “protected zone” during anatomical enucleation—holds value for wider adoption and offers practical feasibility for surgical training. Compared with some functional preservation techniques requiring specialized equipment (such as holmium lasers), FP-PKEP relies entirely on the bipolar plasma platform, offering greater accessibility in primary healthcare settings. Future research should proceed in the following directions: (1) conducting prospective multicenter randomized controlled trials (RCTs) comparing FP-PKEP with conventional PKEP, using the urinary continence rate and the rate of preserved ejaculatory function at 6 months postoperatively as co-primary endpoints; (2) employing validated instruments, such as the MSHQ-EjD-SF, for the standardized assessment of ejaculatory function; (3) extending follow-up to at least 3 years to evaluate the durability of urinary function, long-term re-intervention rates, and overall changes in quality of life; and (4) investigating objective mechanical evidence regarding how the preservation of urethral mucosa at the 12 o'clock position improves urinary continence, utilizing videourodynamics or magnetic resonance imaging (MRI).

## 5 Conclusions

This single-center retrospective case series suggests that FP-PKEP may achieve improvements in voiding function comparable to those of existing EEP techniques in patients with large-volume BPH, together with promising early recovery of urinary continence (transient incontinence rate of 7.7%, with all cases resolving within one month) and preservation of antegrade ejaculation (clinically suspected retrograde ejaculation rate of 12.5%). The systematic preservation of the bladder neck circular fibers and of the urethral mucosa at the 12 o'clock position represents an anatomically grounded and technically feasible modification of the standard PKEP technique. However, because this was an uncontrolled case series with a small sample size and short follow-up, these findings are exploratory and do not establish superiority over conventional PKEP or other EEP techniques; validation through adequately powered randomized controlled trials is required before the technique can be recommended for routine clinical use.

## Data Availability

The raw data supporting the conclusions of this article will be made available by the authors, without undue reservation.
